# Enhancing Primaquine Adherence for *Plasmodium vivax* Malaria: A Cluster-Randomized Controlled Trial in Myanmar

**DOI:** 10.4269/ajtmh.24-0814

**Published:** 2025-05-06

**Authors:** Kyawt Mon Win, Pyae Linn Aung, Nay Yi Yi Linn, Zau Ring, Myat Phone Kyaw, Wang Nguitragool, Liwang Cui, Jetsumon Sattabongkot, Saranath Lawpoolsri

**Affiliations:** ^1^ Department of Tropical Hygiene, Faculty of Tropical Medicine, Mahidol University, Bangkok, Thailand;; ^2^ Mahidol Vivax Research Unit, Faculty of Tropical Medicine, Mahidol University, Bangkok, Thailand;; ^3^ Department of Public Health, Ministry of Health, Naypyidaw, Myanmar;; ^4^ State Public Health Department, Kachin, Myanmar;; ^5^ Myanmar Health Network Organization, Yangon, Myanmar;; ^6^ Department of Molecular Tropical Medicine and Genetics, Faculty of Tropical Medicine, Mahidol University, Bangkok, Thailand; and; ^7^ Division of Infectious Diseases and International Medicine, Department of Internal Medicine, Morsani College of Medicine, University of South Florida, Tampa, Florida

## Abstract

The rising prevalence of *Plasmodium vivax* (*P. vivax*) malaria challenges elimination efforts, particularly in the Greater Mekong Subregion, although adherence to the required 14-day primaquine (PQ) regimen remains a major obstacle. This study evaluated the effectiveness of a family-administered, directly observed treatment intervention in improving adherence to PQ among patients with confirmed *P. vivax *from October 2022 to March 2023. A cluster-randomized controlled trial was conducted in five intervention villages and five control villages in Kachin State, Myanmar. A total of 427 patients in the intervention group (supervised dosing) and 425 patients in the control group (unsupervised dosing), all diagnosed with *P. vivax*, underwent a 14-day PQ regimen, with the intervention group being supervised by trained family members. Pill counts on day 14 were assessed and compared between the two groups using Poisson generalized linear mixed models. Parasite reappearance identified by polymerase chain reaction was compared between the two groups using survival analysis. Cumulative malaria incidence at baseline, as well as at months 6 and 12, was compared between the two groups. Treatment adherence was significantly higher in the intervention group (98.8%) compared with the control group (77.6%). Parasite reappearance rates were similar between the groups (*P* = 0.20) on days 14, 28, and 42. However, the cumulative incidence of malaria over 1 year was significantly lower in the intervention group (*P* <0.001) compared with the control group. In malaria-endemic areas with limited resources, a family-administered, directly observed treatment intervention offers an efficient approach to enhance PQ adherence and achieve the radical cure of *P. vivax* malaria.

## INTRODUCTION

*Plasmodium vivax *(*P. vivax*) malaria remains a major public health concern in the Greater Mekong Subregion, as indicated by the World Malaria Report 2023.^1^ In Myanmar, *P. vivax* infections accounted for 81.1% of the 157,538 total malaria cases reported in 2022. The burden of *P. vivax* malaria began to rise in 2019, reversing the previous trend dominated by *Plasmodium falciparum*.^1^ The majority of *P. vivax* cases are concentrated in the border regions of China–Myanmar and Thailand–Myanmar. The Myanmar National Strategic Plan for Malaria Elimination (2021–2025) emphasizes the need to prioritize activities related to the radical cure of *P. vivax*, particularly by promoting adherence to hypnozoitocidal drugs among patients.^2^ According to the current treatment guidelines in Myanmar, uncomplicated *P. vivax* malaria is treated with chloroquine (CQ) and a 14-day course of primaquine (PQ) at a dosage of 0.25 mg/kg/day, without glucose-6-phosphate dehydrogenase (G6PD) testing, and is administered by basic healthcare staff in public health facilities and trained malaria volunteers in community settings.^3^

The radical cure of *P. vivax* malaria requires a combination therapy targeting both blood- and liver-stage parasites to eliminate all parasite reservoirs.^4^ However, adherence to the 14-day PQ regimen is notoriously poor.^5^ Patients who fail to complete the treatment are prone to resurgence or relapse. Factors contributing to the resurgence or relapse of *P. vivax* infections include age, sex, inadequate PQ dosages, low drug concentrations, and high parasitemia.^6^^,^^7^ Moreover, numerous studies have highlighted various factors that determine PQ treatment adherence, including patient-related factors such as ethnicity, inadequate knowledge, and forgetfulness, as well as the perceptions and experiences of healthcare providers.^8^^,^^9^ Side effects of PQ and the rapid effectiveness of CQ also contribute to adherence.^10^^,^^11^ Improving patient adherence to the full treatment course is vital for preventing relapses and ultimately achieving the radical cure of *P. vivax* malaria.^12^

Treatment adherence refers to a patient’s ability to follow prescribed medication instructions.^13^ Several methods are used to measure treatment adherence, including pill counting, patient interviews, self-reporting, and drug concentration measurements.^14^ To bolster treatment adherence, interventions such as health education, provider training, reminder calls and messages, and incentives can be implemented. Additionally, totally or partially supervised treatment has been shown to enhance PQ efficacy.^15^^–^^17^ However, implementing these interventions requires integrating them into the existing healthcare system while ensuring the availability of necessary resources, infrastructure, and support mechanisms.^18^ Currently, the National Malaria Control Program (NMCP) in Myanmar lacks a strategy to promote PQ treatment adherence. This study represents the first interventional study aimed at improving adherence to the radical cure treatment of *P. vivax* malaria in Myanmar.

We previously conducted a qualitative study in a township in northern Myanmar, an area with one of the highest reported cases of *P. vivax*, to characterize the study township and the challenges associated with ensuring patient adherence to the 14-day PQ regimen.^19^ Potential interventions identified included direct observation by family members, shortening the treatment regimen, expanding the volunteer network, and strengthening behavioral change communication. Consequently, we developed and implemented an intervention package that incorporates a family-administered, directly observed treatment (DOT) approach. This present study aims to evaluate the effectiveness of this intervention package in improving adherence to PQ treatment among patients with uncomplicated *P. vivax* malaria.

## MATERIALS AND METHODS

### Study site.

This study was conducted in *P. vivax* malaria-endemic villages in Waingmaw Township, Kachin State, located in northern Myanmar. In 2021, Kachin State accounted for 28% of the *P. vivax* malaria cases in the country. According to data from the NMCP in 2021, Waingmaw Township reported the highest *P. vivax* caseload in Kachin State and experienced an unusual increase in malaria cases during 2020 and 2021. Malaria prevention and control activities in the township are administered collaboratively by the Township Public Health Department and other malaria-implementing partners. Because of the high malaria endemicity, malaria diagnosis and treatment are primarily managed by trained integrated community malaria volunteers (ICMVs) from the NMCP and collaborative partners at the village level.

### Study design.

**Figure 1.**
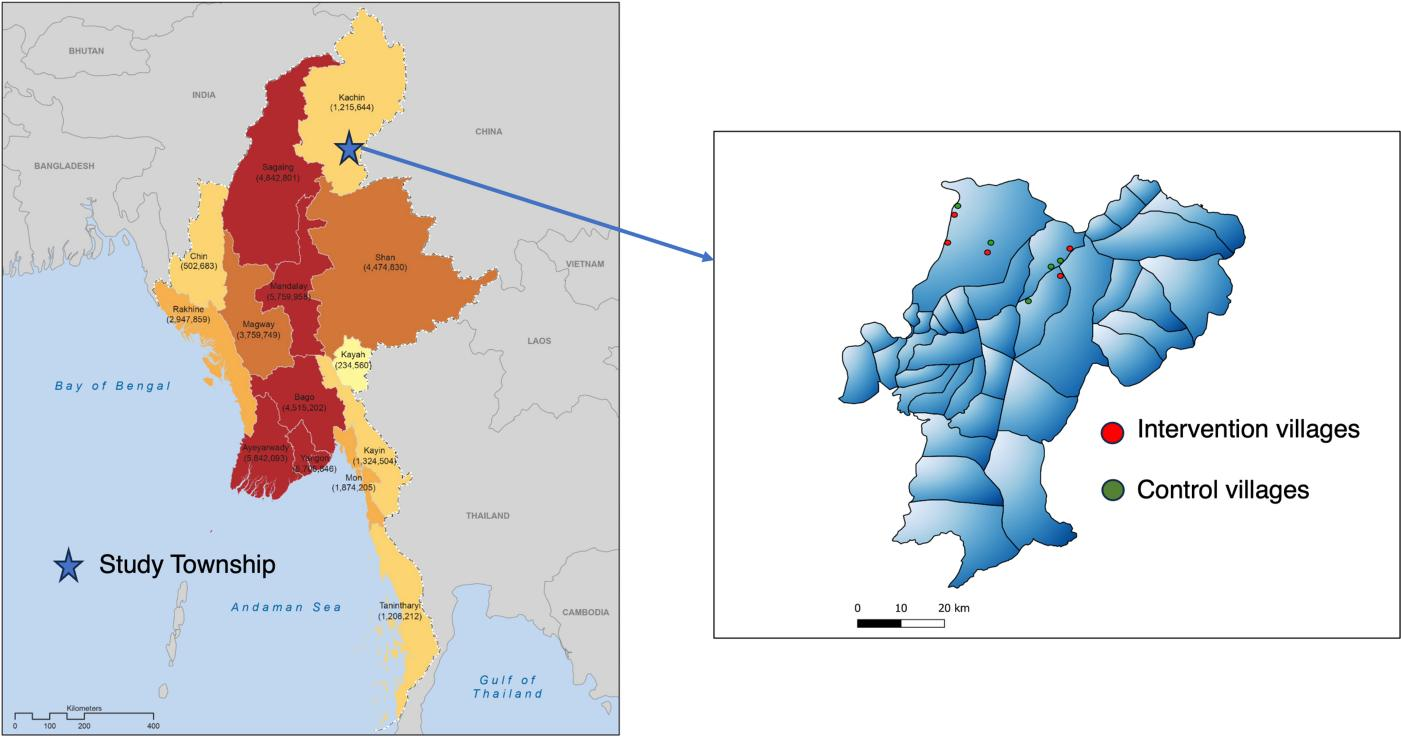
Study township and villages in Waingmaw Township, Kachin State, Myanmar.

A clustered randomized controlled trial was conducted from October 2022 to March 2023. First, we purposively selected 20 of 213 villages in Waingmaw Township with high *P. vivax* prevalence. These 20 villages collectively accounted for 80% of the total reported *P. vivax* cases in the township in 2021. All selected villages shared similar seasonal malaria trends, endemicity levels, sociodemographic characteristics of malaria patients, environmental conditions, and geographical accessibility. The 20 villages were then randomly assigned (1:1) into two groups of 10 clusters: five villages for the intervention group and five villages for the control group. The names of these village clusters were listed and randomly assigned to either the intervention or control group. Because of the nature of the intervention, study participants were not blinded to their allocated status. To minimize contamination, a buffer zone of at least 2 kilometers was established around each selected village (Figure 1). Integrated community malaria volunteers from the intervention clusters underwent specialized training for the intervention. The intervention villages received the developed intervention package alongside standard malaria treatment for *P. vivax* patients, whereas patients in the control group received standard malaria treatment only, which involved self-administration of PQ for 14 days.

### Study participants.

The participants in this study included confirmed uncomplicated *P. vivax* patients aged over 1 year from the selected study villages who consented to participate. Individuals who were ineligible to receive PQ, such as pregnant or lactating women, patients with a history of G6PD deficiency, and individuals with severe *P. vivax* infection were excluded.

### Sample size.

The required sample size was calculated using the standard formula for a cluster-randomized controlled study^20^ to detect differences in 14-day PQ adherence proportions between the intervention and control groups. Initially, the sample size required for an individual randomized trial (1:1 ratio) was calculated. Based on a previous study in Myanmar, in which 46% of patients completed the 14-day PQ regimen,^21^ we assumed that adherence would increase 1.5-fold with the intervention package (70% adherence). Using an alpha error of 0.05, a power of 0.8, and a 1:1 ratio, the sample size required was 66 participants per group. Next, the sample size was adjusted for clustered data with a fixed number of five clusters per group and an intraclass correlation coefficient of 0.01. After accounting for a 10% dropout rate, the adjusted sample size was 84 participants per cluster. Therefore, the total number of participants required for the 10 clusters was 840, with at least 420 participants in each group.

### Intervention.

Based on qualitative findings,^19^ we developed a feasible intervention package selected from several potential interventions. This package was aligned with the activities outlined in the Myanmar National Strategic Plan and the National Malaria Elimination Guidelines.^2^^,^^22^ The intervention emphasized family-administered directly observed therapy (family DOT), in which anti-malaria drugs were administered under the supervision of a family member. Similar approaches have demonstrated success in anti-tuberculosis medication programs.^23^

To implement family DOT, five ICMVs from the selected villages were trained. These ICMVs included one male and four female participants aged between 40 and 65 years, all of whom had completed tertiary education. The training emphasized the importance of radical cure for *P. vivax*, the duration of treatment, the management of adverse effects, and the role of family members in administering family DOT. The training was conducted by the study team at the Township Public Health Department and included demonstrations and practice sessions.

When a *P. vivax* patient was diagnosed via rapid diagnostic test (RDT), trained ICMVs provided on-site training to the patient’s family members or accompanying individuals regarding the family DOT procedure. There were no specific education or occupation requirements for family members to become DOT providers. The DOT provider administered the daily prescribed doses of CQ for 3 days and PQ for 14 days directly in front of the patient at a designated time. According to Myanmar’s national malaria treatment guidelines,^3^ G6PD testing was not required before providing PQ. Additionally, standardized pamphlets containing health messages and guidance for family DOT, which reinforce key messages on *P. vivax* radical cure, were distributed to patients and their companions. These pamphlets were developed by the study team under the guidance of malaria experts from the NMCP and were designed to reinforce key messages about the radical cure of *P. vivax*. The content aligned with current malaria treatment guidelines to ensure treatment adherence.

### Control.

Patients with confirmed uncomplicated *P. vivax* infections in the control clusters underwent the standard treatment, which consisted of 3 days of CQ along with a 14-day regimen of PQ at a dosage of 0.25 mg/kg/day, in accordance with national treatment guidelines. Standard instructions were provided to patients to complete the full treatment regimen without additional supervision. Patients from both the intervention and control groups were instructed to retake the dose if they vomited within 30 minutes of taking it to ensure sufficient absorption.

### Data collection.

Trained ICMVs from each selected village served as focal points for implementing the intervention and collecting data. Malaria infections were confirmed using the SD Bioline Malaria Antigen *P.f*/*P.v* RDT (Abbott Laboratories, Abbott Park, IL). Patients diagnosed with uncomplicated *P. vivax* infections were enrolled. Clinical and treatment data were recorded using the routine malaria case report form. In addition, other socioeconomic factors, such as education, occupation, family income, and history of malaria, were documented using a structured case record form.

### Assessment of adherence.

The primary outcome of the study was treatment adherence, assessed through a pill count on day 14. Structured surveys were also conducted to record side effects and other relevant factors, such as the characteristics of DOT providers. A standardized urine color chart was provided to the participants to determine changes in urine color. A patient was considered adherent if no PQ tablets remained by day 14. The presence of any remaining pills indicated nonadherence.

### Assessment of parasitemia after treatment.

In addition to the pill counts and quantitative questionnaires, finger-prick blood samples were collected in dried blood spots (100 microliters) for molecular confirmation of parasitemia. This included the analysis of the 18S ribosomal RNA genes using nested polymerase chain reaction (PCR) and genotyping of the polymorphic *P. vivax* merozoite protein 1 gene. Blood samples were collected on days 0, 14, 28, and 42 from patients in both groups. Additional samples were collected on day 7 in the intervention group to assess early treatment failure, whereas day 7 samples were omitted from the control group to avoid routine treatment interference. Percentages of parasitemia on days 14, 28, and 42 were compared between groups. Home visits were conducted by trained ICMVs under the supervision of the study team. Given that many patients were forest-goers or mobile migrant workers, finger-prick blood samples were only collected from local patients or permanent village residents. This approach ensured consistent blood collection from individuals throughout the 42-day follow-up, given the resource constraints.

### Assessment of malaria incidence.

The secondary outcome was the cumulative malaria incidence in the intervention and control groups. Cumulative incidences were calculated using data extracted from the electronic national malaria reporting system for each village. In this system, individual case-based data for all reported malaria cases were collected on a monthly basis. Cumulative incidences were analyzed at three time points: the start of the intervention, 6 months after the intervention, and 1 year after the intervention. According to the electronic national malaria reporting system, all reported cases were treated in accordance with national treatment guidelines.

## STATISTICAL ANALYSES

Data were initially entered into Microsoft Excel (Microsoft Corp., Redmond, WA) and analyzed using R statistical software (version 2022.12.0 + 353; R Core Team 2023, Vienna, Austria).^24^ χ^2^ tests were performed to detect differences in participant characteristics and side effects between groups. The proportions of PQ adherence among reported *P. vivax* patients were calculated for both groups. RRs and their 95% CIs were calculated to identify factors associated with treatment adherence, with adjustments made for cluster effects using a Poisson generalized linear mixed model. Only variables that showed a *P*-value of less than 0.2 in the simple regression were included in the multivariate models to validate the magnitude of associations. The proportion of participants with parasitemia at each assessment period (days 14, 28, and 42) was calculated. Survival analysis was conducted to compare parasite reappearance rates between the intervention and control groups. Cumulative malaria incidence was calculated as the total number of malaria cases reported each month divided by the total population in the intervention or control group. Cumulative incidences at baseline, 6 months, and 12 months after the intervention were compared between the two groups by calculating cumulative incidence differences and cumulative incidence ratios, along with their 95% CIs.

## RESULTS

### Demographic characteristics of participants.

**Figure 2.**
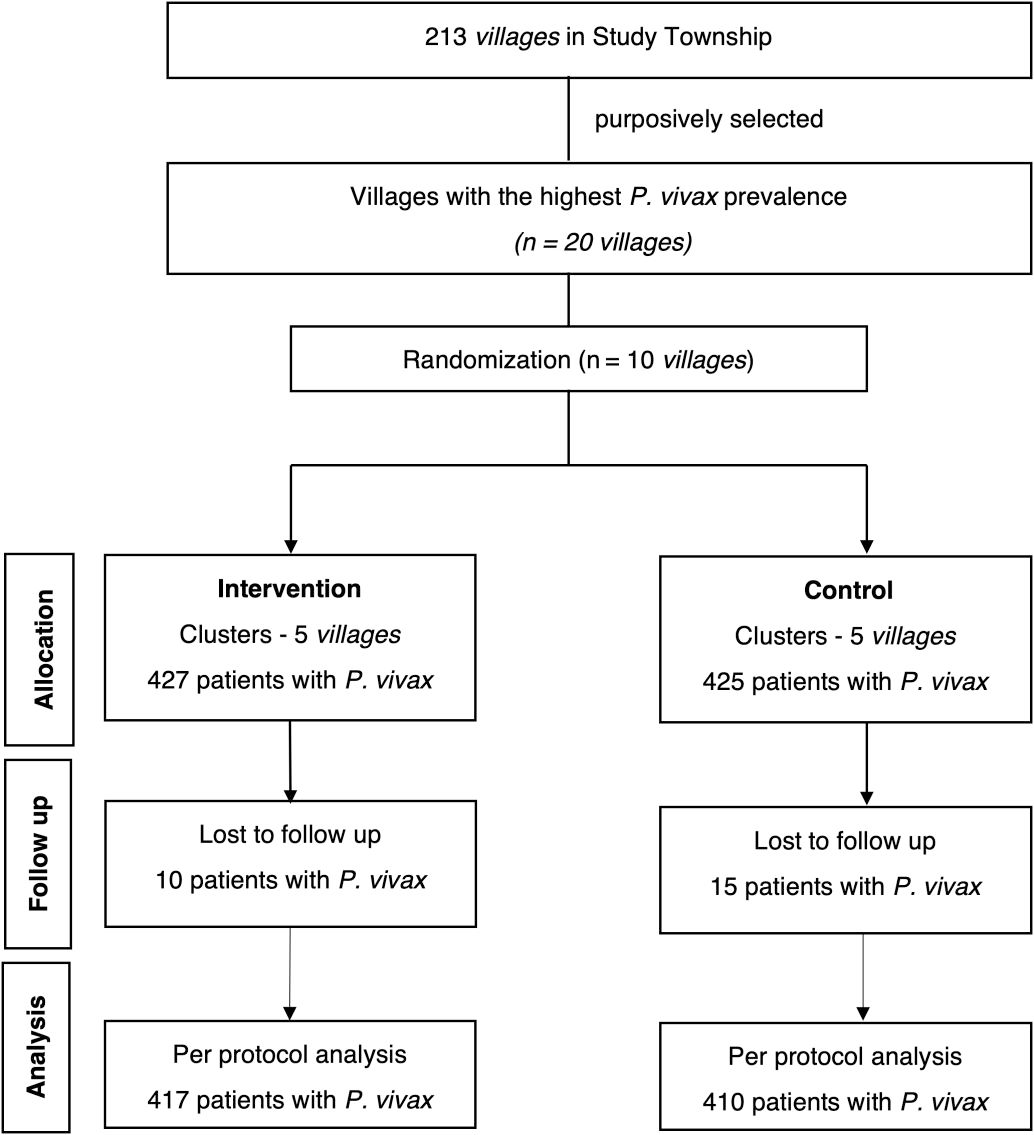
CONSORT flow diagram.

Between October 2022 and March 2023, a total of 852 *P. vivax* patients were screened and enrolled in the study, with 427 participants assigned to the intervention groups across five clusters and 425 patients assigned to the five clusters in the control group. Twenty-five participants (10 in the intervention group and 15 in the control group) were lost to follow-up and were excluded from further analysis of adherence (Figure 2).

The demographic characteristics of participants were compared between the two groups in Table 1. Approximately half of the participants in each group were aged 5 to 9 years and 15 to 30 years. In the intervention group, more than two-fifths of the participants were engaged in forest-related work (41.2%) and had completed primary education (42.2%). More than half (55.0%) had a monthly family income of 100,000–300,000 Myanmar kyat (MMK). Similarly, in the control group, more than one-third (35.5%) were engaged in forest-related work, about half (50.8%) had completed primary education, and the majority (60.2%) had a monthly family income of 100,000–300,000 MMK. Forest-related occupations were identified as the primary sources of income in the study village, where children aged 10 years and above often worked alongside their families, either in the forest or within the village. Furthermore, approximately one-third of participants in both groups (36.3% in the intervention and 40% in the control group) had a history of malaria. There were no significant differences in demographic or background characteristics between the two groups (*P* >0.05).

**Table 1:** Demographic characteristics of study participants

Characteristics	Intervention	Control	*P*-Value
	(*n* = 427)	(*n* = 425)	
	*N* (%)	*N* (%)	
Age (years)			
1–4	61 (14.3)	47 (11.1)	0.309
5–9	103 (24.1)	114 (26.8)	–
10–14	62 (14.5)	81 (19.1)	–
15–30	110 (25.8)	104 (24.5)	–
31–60	85 (19.9)	73 (17.1)	–
>60	6 (1.4)	6 (1.4)	–
Sex			
Male	225 (52.7)	196 (46.1)	0.064
Female	202 (47.3)	229 (53.9)	–
Occupation			
Children (1–9 years)	164 (38.4)	161 (37.9)	0.070
Forest-related	176 (41.2)	151 (35.5)	–
Non-forest-related	87 (20.4)	113 (26.6)	–
Education			
Children (1–4 years)	61 (14.3)	47 (11.1)	0.097
Illiterate	4 (0.9)	6 (1.4)	–
Primary education	180 (42.2)	216 (50.8)	–
Secondary education	155 (36.3)	129 (30.4)	–
Higher education	27 (6.3)	27 (6.4)	–
Monthly family income (MMK)			
Less than 100,000	85 (19.9)	69 (16.2)	0.319
100,000–300,000	239 (55.9)	256 (60.2)	–
More than 300,000	103 (24.2)	100 (23.6)	–
Past malaria history			
Yes	155 (36.3)	170 (40)	0.298
No	272 (63.7)	255 (60)	–

MMK = Myanmar kyat ($1 = ∼3,000 MMK).

### Proportion of PQ treatment adherence.

All participants in both groups were tested and treated by ICMVs from their respective villages. The primary outcome, treatment adherence, was assessed based on the patients’ ability to follow prescribed medication instructions. Initially, all participants completed the 3-day CQ treatment regimen as prescribed. However, adherence to the 14-day PQ treatment was significantly higher in the intervention group (98.8%) compared with the control group (77.6%; *P* <0.001). Pill count data indicated that the majority (96.7%) of nonadherence cases had remaining PQ tablets for up to a maximum of a 7-day dose. However, the specific day of treatment on which nonadherence occurred could not be determined. Reasons for nonadherence among the 98 participants who did not complete the full treatment course included forgetting to take the medication (62.2%), symptom relief within the first 3–4 days (21.4%), and the occurrence of side effects (16.3%).

### Relative side effects.

**Figure 3.**
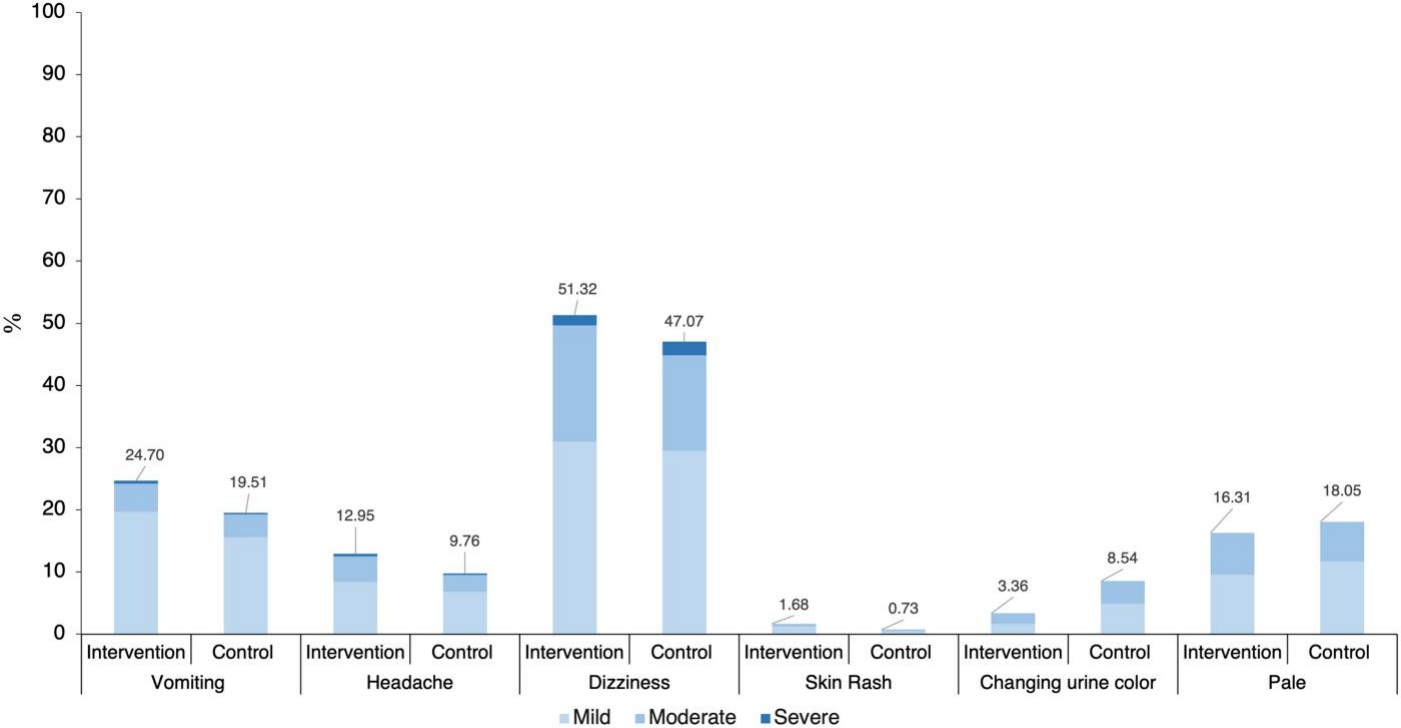
Proportion of relative adverse effects with severity levels of antimalarial drugs.

Approximately half of the participants (58.5% in the intervention group and 54.1% in the control group) experienced adverse effects during treatment, with most of the symptoms ranging from mild to moderate in severity (Figure 3). Dizziness was the most commonly reported adverse effect (49.2% of all cases), whereas severe symptoms were rare (2–3%). Some of the participants (nearly one-fourth in both groups) experienced hemolytic-related adverse effects, such as anemia. There was no significant difference in adverse effects between the intervention and control groups (*P* >0.05), except for urine color changes, which were more frequent in the control group (3.36% in the intervention group and 8.54% in the control group; *P* = 0.003).

### Directly observed treatment providers (intervention group only).

Of 417 participants, 252 (55.6%) received treatment from their parents, 54 (12.9%) from their spouses, and 49 (11.7%) from their siblings. The remaining patients received treatment from various sources, including relatives (2.3%) and friends (8.2%). A few patients (9.4%) living alone in the forest self-administered antimalarial drugs, with three participants being nonadherent. Among the 378 DOT providers, more than two-fifths (40.4%) had a history of malaria. Additionally, most of the providers (81.0%) were engaged in forest-related occupations.

### Factors associated with PQ adherence.

According to the Poisson regression model, patients in the intervention group showed significantly higher adherence than those in the control group (adjusted RR = 1.11; 95% CI: 1.01–1.23). However, PQ adherence was not significantly associated with variables such as history of malaria, family income, sex, adverse effects, occupation, or education (Table 2).

**Table 2:** Predictors for treatment adherence among patients with *Plasmodium vivax*

No.	Characteristics	Adherence, *N* (row %)	Nonadherence, *N* (row %)	cRR (95% CI)	*P*-Value	aRR (95% CI)
1.	Groups					
	Control	317 (77.3)	93 (22.7)	Ref.	–	Ref.
	Intervention	409 (98.1)	8 (1.9)	1.11 (1.01–1.23)	<0.001*	1.11 (1.01–1.23)
2.	Age (years)					
	1–4	82 (75.9)	26 (24.1)	Ref.	–	–
	5–9	197 (93.4)	14 (6.6)	1.1 (0.9–1.31)	0.3	–
	10–14	115 (81.6)	26 (18.4)	1.03 (0.86–1.25)	0.7	–
	15–30	183 (86.7)	28 (13.3)	1.06 (0.89–1.26)	0.5	–
	>30	149 (95.5)	7 (4.5)	1.11 (0.92–1.33)	0.3	–
3.	Sex					
	Male	365 (89.9)	41 (10.1)	Ref.	–	–
	Female	361 (85.7)	60 (14.3)	0.98 (0.89–1.08)	0.66	–
4.	Occupation					
	Non-forest-related	449 (86.5)	70 (13.5)	Ref.	–	–
	Forest-related	277 (89.9)	31 (10.1)	1.01 (0.92–1.13)	0.72	–
5.	Past malaria history					
	No	493 (95.4)	24 (4.6)	Ref.	–	Ref.
	Yes	233 (75.2)	77 (24.8)	0.9 (0.8–0.99)	0.04*	0.9 (0.82–1.02)
6.	Adverse effect					
	No	331 (91.4)	31 (8.6)	Ref.	0.5	–
	Yes	395 (84.9)	70 (15.1)	0.96 (0.87–1.06)	–	–
7.	Education					
	Illiterate	89 (87.3)	13 (12.7)	Ref.	–	–
	Primary	342 (87.5)	49 (12.5)	1 (0.85–1.17)	0.99	–
	Secondary	247 (87.9)	34 (12.1)	1.01 (0.85–1.18)	0.95	–
	Higher	48 (90.6)	5 (9.4)	1.02 (0.81–1.27)	0.88	–
8.	Monthly family incomes (MMK)					
	Less than 100,000	117 (78.0)	33 (22.0)	Ref.	–	Ref.
	100,000–300,000	459 (95.6)	23 (4.8)	1.13 (1–1.29)	0.06	1.1 (0.97–1.27)
	More than 300,000	152 (77.2)	45 (22.8)	1 (0.79–1.28)	0.9	1.01 (0.79–1.28)

aRR = adjusted relative risk; cRR = crude relative risk; MMK = Myanmar kyat. Generalized linear mixed model fit by maximum Family: Poisson (log).

* Significance at *P*-value <0.05.

### Parasite reappearance.

**Figure 4.**
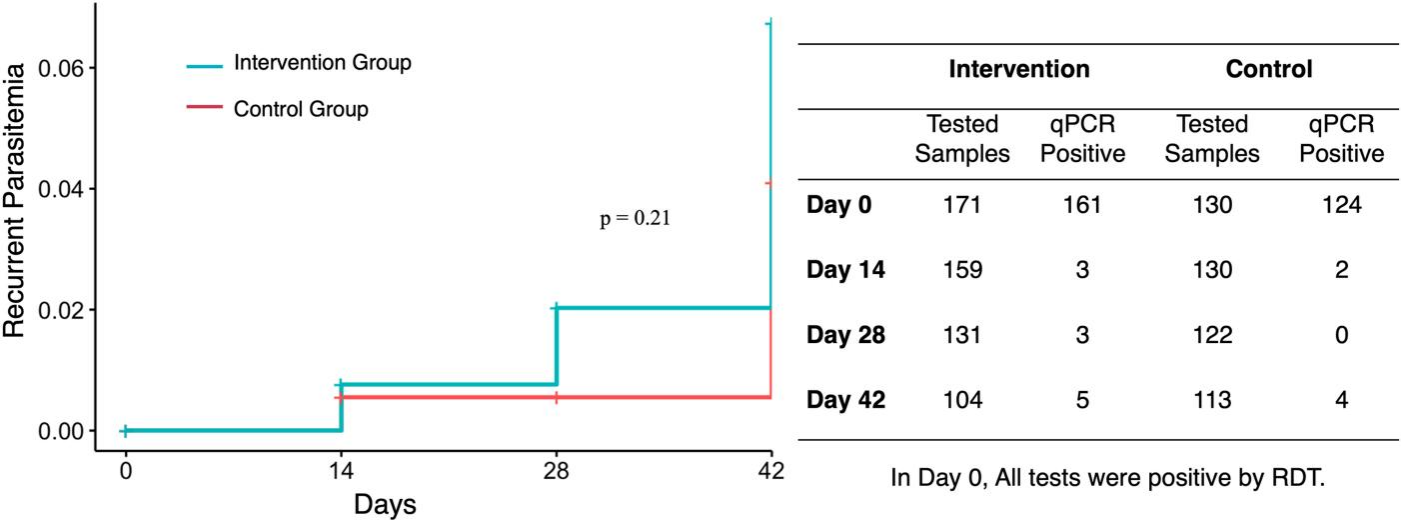
Survival analysis curve of parasite reappearance.

A total of 1,226 samples were collected from 301 patients residing in the local villages, including 171 from the intervention group and 130 from the control group. All samples were diagnosed as *P. vivax* by RDT. Cumulative parasitemia reappearance rates were 0.007 and 0.006 on day 14 in the intervention and control groups, respectively. By days 28 and 42, the reappearance rates increased to 0.02 and 0.07 in the intervention group and 0.006 and 0.04 in the control group, respectively. However, these differences were not statistically significant (*P* = 0.21; log-rank test; Figure 4).

### Malaria cumulative incidence.

**Figure 5.**
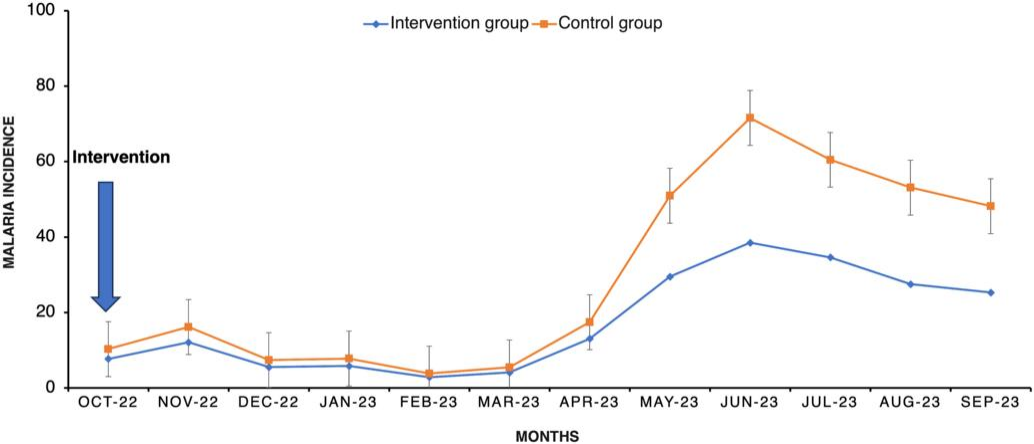
Monthly malaria cumulative incidence (cases per 1,000 person-months) of intervention and control villages.

A comparative analysis of monthly malaria cumulative incidence was conducted pre- and post-intervention for both groups. Initially, the baseline monthly malaria cumulative incidence was 7.69 (95% CI: 5.39–10.65) in the intervention group and 10.25 (95% CI: 6.5–15.38) in the control group, with a difference of 2.56. A subsequent assessment 1 year post-intervention revealed a different scenario; the cumulative incidence increased to 23.08 (95% CI: 18.93–27.87) in the intervention group, whereas it surged to 52.58 (95% CI: 43.53–62.97) in the control group, resulting in a difference of 29.5. This increase indicated a significant difference in the reduction of malaria cumulative incidence between the groups (Figure 5 and Table 3).

**Table 3:** Comparison of monthly malaria cumulative incidence between intervention and control groups

Time	Cumulative Incidence Risk		IRD	IRR	*P*-Value
	Intervention Group	Control Group			
Baseline	7.69 (5.39 to 10.65)	10.25 (6.5 to 15.38)	2.56 (–2.2 to 8.3)	0.75 (0.43 to 1.32)	0.2
6 months after intervention	13.04 (9.97 to 16.75)	20.5 (14.6 to 26.83)	7.46 (1.2 to 14.8)	0.65 (0.43 to 0.97)	0.03*
1 year after intervention	23.08 (18.93 to 27.87)	52.58 (43.53 to 62.97)	29.5 (19.8 to 40.3)	0.44 (0.33 to 0.57)	<0.001*

IRD = incidence risk difference (intervention versus control); IRR = incidence risk ratio (intervention versus control).

* Significance at *P*-value <0.05.

## DISCUSSION

The DOT approach has been shown to improve drug adherence in numerous studies worldwide, addressing major diseases such as tuberculosis and malaria.^16^^,^^17^^,^^25^^,^^26^ This approach comprises, but is not limited to, administering DOT by healthcare providers and family members. Especially noteworthy is DOT by family members, which not only ensures effective drug adherence but also proves cost-effective, saving on operational expenses such as travel and human resources while overcoming transportation issues that may otherwise arise.^15^^,^^25^^,^^26^ Findings from this study confirm that DOT can effectively increase adherence to the 14-day PQ treatment among patients with vivax malaria. Similarly, in this study, accompanied persons—whether family members, friends, relatives, or parents—were enlisted as drug providers after receiving standardized pamphlets that emphasized the importance of completing the treatment regimen. This approach has been shown to improve 14-day PQ adherence^27^ and could be scaled up as a national-level strategy with situation-specific adjustments made by technical bodies such as the WHO.

One of the major factors contributing to poor adherence to the 14-day PQ course is its long duration.^28^ According to a study in Thailand, only 23.8% of patients with *P. vivax* exhibited complete adherence to the 14-day PQ regimen.^8^ Although overall adherence percentages were relatively high in our study, adherence was particularly high up to 1 week, especially in the control group. Several studies have demonstrated the effectiveness of shorter PQ regimens, such as a 7-day treatment option, whose efficacy is comparable to that of the traditional 14-day course.^28^^,^^29^ National programs may consider piloting such shorter regimens with G6PD testing in the future. Reasons for not completing the full treatment course mainly included forgetfulness and relief from symptoms.^19^ Typically, once individuals experience symptom relief, they may be reluctant to continue medication, possibly saving it for future use or sharing it with family members or friends.^30^ In our study, despite briefings given to accompanying persons to remind patients to take their medicines regularly and completely, occasional lapses occurred because of forgetfulness or other personal and household responsibilities. Directly educating patients about the importance of full compliance with treatment may also prove helpful in future interventions.

Primaquine is contraindicated for individuals such as pregnant and breastfeeding mothers, newborns, and those with very severe G6PD deficiency due to the risk of hemolytic anemia.^31^ However, routine testing of G6PD for every *P. vivax* patient before treatment is not yet implemented in Myanmar because of resource limitations, including shortages of healthcare workers and budget constraints. Clinical trials for PQ have shown a diminished occurrence of side effects, both in terms of frequency and severity.^32^ In this study, however, hundreds of patients with *P. vivax* were treated with a 14-day course of PQ, and inquiries into the presence of side-effect-like symptoms were conducted as part of the follow-up. Many patients reported symptoms such as dizziness (49.2%) and vomiting (22.2%), whereas a few mentioned paleness (1.2%) and changes in urine color (5.9%). Although no severe cases were identified in this study, it was difficult to determine whether the expressed symptoms were related to either CQ or PQ. Close observation of patients during PQ treatment is necessary. Among several studies documenting G6PD deficiency prevalence ranging from 5.6% to 19.8% in Myanmar,^33^^–^^36^ ensuring the completeness of drug administration should be balanced with precautions for the safety of every patient. As part of the intervention in future implementations, it may be possible to request that persons accompanying patients observe any unusual hemolytic symptoms, such as red-colored urine, during DOT.

One modeling study from Thailand attributed nearly 79% of *P. vivax* cases to relapses rather than new infections.^37^ The latent hypnozoite form of *P. vivax* parasites can be reactivated over varying periods, ranging from days to years.^38^ According to routine national surveillance data from the study township, the intervention group exhibited improvement over time, although the overall malaria trend did not show a sharp decline. This improvement may be attributed to the hypnozoidal activity of PQ following good adherence. Currently, the 14-day course of PQ is the sole viable option for achieving a radical cure of *P. vivax* in Myanmar, whereas other 8-aminoquinolones, such as tafenoquine, are still in clinical trials.^39^ The reappearance of *P. vivax* parasitemia was observed in both the intervention and control groups, though there was no significant difference. A study conducted in the Thailand–Myanmar border areas also concluded that the vivax reappearance rate was significantly lower in the DOT group compared with the self-administered group.^17^ Unfortunately, the present study lacks results from further genotyping diagnostics to confirm whether these reappearing parasites resulted from relapses or reinfections. The relationship between adherence and treatment success in terms of preventing relapses requires further investigation. The study township, one of the highest malaria burden hotspots in Myanmar, is home to highly competent malaria vectors and significant population movements, including internal displacements.^40^^,^^41^ Continuous active transmission occurs throughout the entire township. Therefore, the reported PCR-positive cases during the follow-up days are most likely attributable to new infections. However, these reappearance events could also result from recurrent infections, as highlighted by a study in Thailand indicating that many *P. vivax* patients experience multiple episodes within a year, with a median interval of 90 days.^42^ In addition to drug adherence, preventive measures such as the use of long-lasting insecticidal nets (LLINs) should be emphasized. Furthermore, studies have shown the potential for CQ resistance in many townships in Myanmar.^43^^,^^44^ Continuous surveillance efforts are needed to monitor possible drug resistance.

This study has both strengths and limitations. To the best of our knowledge, this is the first research initiative piloting an intervention to enhance adherence to PQ in Myanmar. Adherence to the drug was measured at the individual level using both a checklist and activities, such as counting the remaining pills, to enhance the reliability of the results. The 1-year follow-up after the intervention aimed to observe the decreasing trend in overall malaria incidence within the intervention group. However, future studies should consider a longer duration of follow-up to track changes over time, given the chance of relapse. Ongoing political conflicts within the country may also impede the effectiveness of the intervention. Conversely, the current intervention approach may address the shortage of the healthcare workforce in Myanmar.^45^ In this study, we documented individual factors conducive to complete adherence to CQ and PQ. Nevertheless, further investigations are needed to better understand the barriers and challenges inhibiting completion of the 14-day PQ course. For example, a qualitative study could include in-depth interviews and focus group discussions with patients who have had *P. vivax* and did not complete the full treatment course. The cumulative parasitemia reappearance rates might have been higher if we had included non-resident participants, such as forest workers who are at higher risk of reinfection. Compared with the control group, the intervention group showed a reduction in incidence, which could be attributed to the intervention. However, this study did not statistically link the intervention to the decrease in overall incidence. Other environmental factors, such as anopheline mosquito density, vector abundance, and concurrent malaria control activities, warrant further investigation.

## CONCLUSION

This study highlights the effectiveness of family-oriented DOT in enhancing PQ treatment adherence for *P. vivax* malaria. Given that the duration of PQ treatment poses a major barrier to adherence, shorter PQ regimens present a potential intervention. However, robust systems for monitoring G6PD deficiency and adverse effects are prerequisites for implementing high-dose, shorter regimens with PQ. Despite the high malaria endemicity in the study area, the intervention notably reduced malaria incidence. However, ongoing conflicts and population displacement have contributed to an increase in malaria cases; therefore, malaria control efforts must be strengthened through preventive practices such as health promotion and the distribution of LLINs. Moving forward, the NMCP should prioritize research areas that address malaria recurrences, the cost-effectiveness of implementing family-administered DOT, and strategies to enhance treatment adherence within Myanmar.
